# Prognostic genes in the tumor microenvironment in cervical squamous cell carcinoma

**DOI:** 10.18632/aging.102429

**Published:** 2019-11-18

**Authors:** Xin-Bin Pan, Yan Lu, Jian-Li Huang, Ying Long, De-Sheng Yao

**Affiliations:** 1 Department of Radiation Oncology, Guangxi Medical University Cancer Hospital, Nanning, Guangxi 530021, P.R. China; 2 Department of Gynecologic Oncology, Guangxi Medical University Cancer Hospital, Nanning, Guangxi 530021, P.R. China

**Keywords:** cervical squamous cell carcinoma, tumor microenvironment, immune scores, TCGA

## Abstract

Cervical squamous cell carcinoma (CSCC) is one of the most commonly occurring gynecological malignancies. Because CSCC is a biologically heterogeneous disease, its prognosis varies. Therefore, identifying prognostic biomarkers that reflect its biological heterogeneity could lead to better interventions for patients with a poor prognosis. This study used the ESTIMATE algorithm to identify immune related prognostic genes within the tumor microenvironment of CSCC. The results revealed that high immune scores were associated with better overall survival (P = 0.029). Differential expression analysis revealed 384 intersection genes influencing both the immune and stromal scores. Gene Ontology and Kyoto Encyclopedia of Genes and Genomes analyses showed the 384 intersection genes to be mainly enriched for T cell activation, the region of the membrane, carbohydrate binding, and cytokine-cytokine receptor interaction. Among them, 149 immune genes were predictive of overall survival in CSCC. These findings provide a more comprehensive understanding of immune genes within the tumor microenvironment as well as a list of immune genes prognostic in CSCC.

## INTRODUCTION

Cervical squamous cell carcinoma (CSCC) is a serious threat to women's health that causes about 273,200 deaths each year [1]. Surgery is recommended for early stage disease, while surgery plus chemotherapy and radiotherapy improves survival in patients with advanced disease [2]. But despite these treatments, the 5-year overall survival remains unsatisfactory, ranging from 55% to 82% [3–5]. The International Federation of Gynecology and Obstetrics (FIGO) stage is the most important prognostic factor for CSCC. However, the FIGO stage does not reflect the biological heterogeneity of CSCC. Patients with the same FIGO stage may have distinctly different treatment outcomes. Thus, identifying prognostic biomarkers that reflect the biological heterogeneity of CSCC could lead to better interventions for patients with an otherwise poor prognosis.

The microenvironment of malignant tumors consists of immune cells, stromal cells, extracellular matrix molecules and inflammatory mediators [6]. The various components of the microenvironment play central roles in the onset, progression and metastasis of CSCC, and therefore have an important impact on clinical outcomes [7–11]. For example, high levels of immune cell infiltration are reportedly associated with better survival [12, 13], and it has been suggested that the activities of both immune and stromal cells may be predictive of prognosis [12–14].

Estimation of STromal and Immune cells in MAlignant Tumor tissues using Expression data (ESTIMATE) was designed to provide scores for the levels of immune cells infiltration and stromal cells within the tumor microenvironment based on the specific gene expression signatures of immune and stromal cells [9]. Several studies have used ESTIMATE to assess immune, stromal and ESTIMATE scores with overall survival [14, 15]. However, previous studies reported conflict results. Whether ESTIMATE algorithm could be used to investigate prognosis in CSCC is still unclear. In the present study, we used the ESTIMATE algorithm to explore potential genetic factors in the tumor microenvironment of CSCC with the aim of identifying prognostic genes of CSCC.

## RESULTS

### Immune, stromal, and ESTIMATE scores were not correlated with T stage, N stage or tumor grade

**Figure 1:**
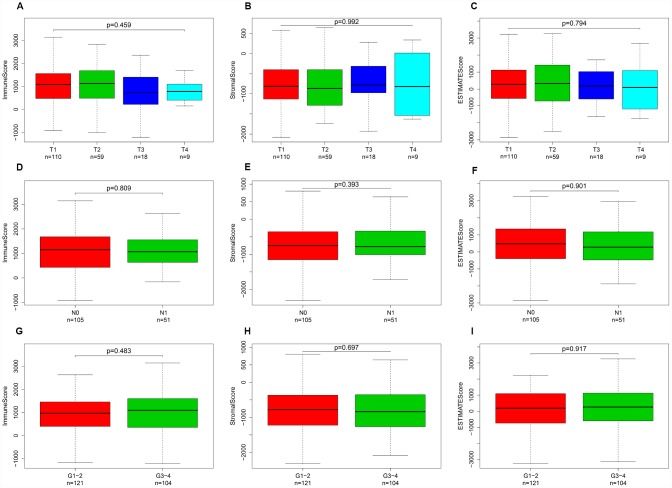
**Immune, stromal, and ESTIMATE scores were not correlated with T stage, N stage, or tumor grade.** Distribution of immune scores plotted against T stage (**A**), N stage (**D**), and tumor grade (**G**). Distribution of stromal scores plotted against T stage (**B**), N stage (**E**), and tumor grade (**H**). Distribution of ESTIMATE scores plotted against T stage (**C**), N stage (**F**), and tumor grade (**I**).

Calculated using the ESTIMATE algorithm, stromal scores ranged from -2318.19 to 804.22 (-806.92 ± 606.45), immune scores ranged from -1209.74 to 3419.33 (950.10 ± 834.53), and ESTIMATE scores ranged from -3262.05 to 4002.00 (143.18 ± 1283.43). The distributions of immune, stromal, and ESTIMATE scores did not vary with T stage (Figure 1A–1C), N stage (Figure 1D–1F), or tumor grade (Figure 1G–1I).

### Elevated immune scores correlate with a better prognosis

**Figure 2:**
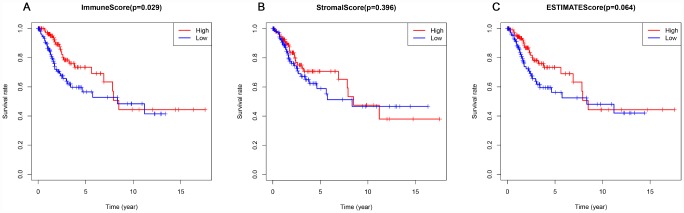
**Association of immune, stromal, and ESTIMATE scores with overall survival.** (**A**) Elevated immune scores correlated with a better prognosis. (**B**) Stromal scores were not associated with overall survival. (**C**) ESTIMATE scores were not associated with overall survival.

**Figure 3:**
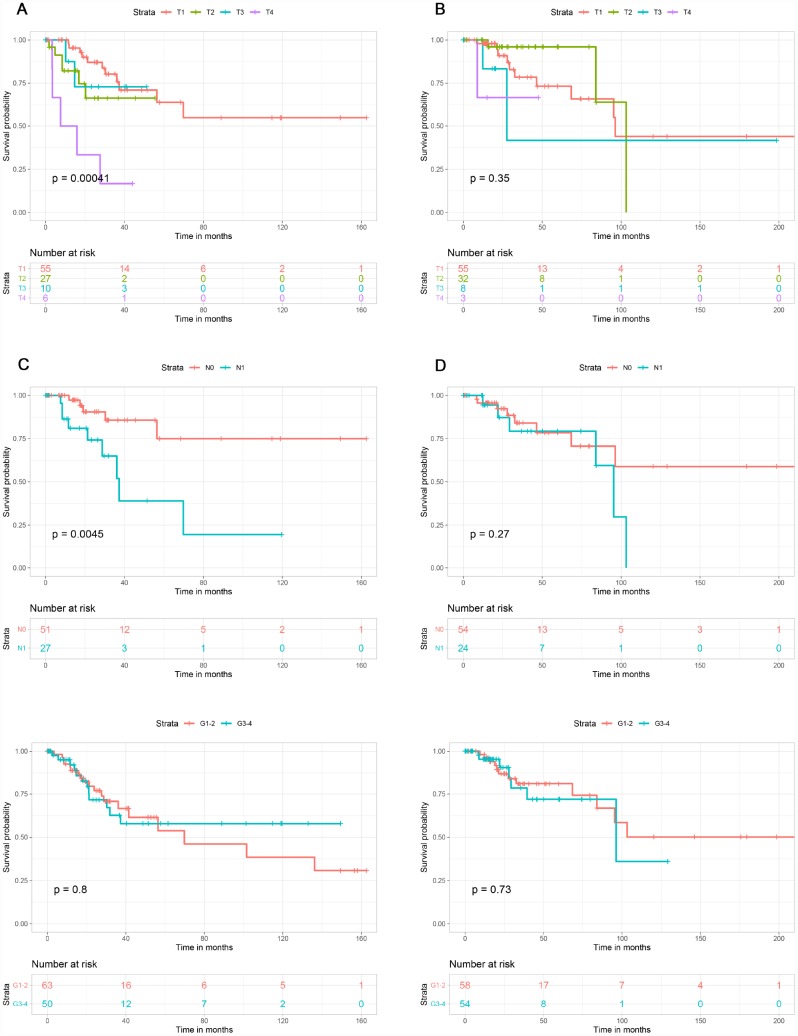
**Association of T stage, N stage and tumor grade with overall survival in the low and high immune score groups.** T stage was associated with overall survival in the low immune score group (**A**), but not the high immune score group (**B**). N stage was associated with overall survival in low immune score group (**C**), but not in high immune score group (**D**). Tumor grade was not associated with overall survival in low (**E**) or high (**F**) immune score group.

This study included 253 CSCC patients. Based on their immune, stromal, and ESTIMATE scores, they were divided into high and low score groups to explore the potential correlation between the scores and prognosis. For immune scores, 127 patients were in the low score group, while 126 were in the high score group. Kaplan-Meier survival curves revealed that elevated immune scores correlated with better overall survival (P = 0.029) (Figure 2A). By contrast, stromal scores (P = 0.396) and ESTIMATE scores (P = 0.064) were not associated with overall survival (Figure 2B, 2C). Further evaluation revealed that T4 stage (P < 0.01) and N1 stage (P < 0.01) were associated with poor overall survival in the low immune score group, whereas there was no association between overall survival and T stage, N stage or tumor grade in the high immune score group (Figure 3).

### Differentially expressed genes with immune and stromal score

**Figure 4:**
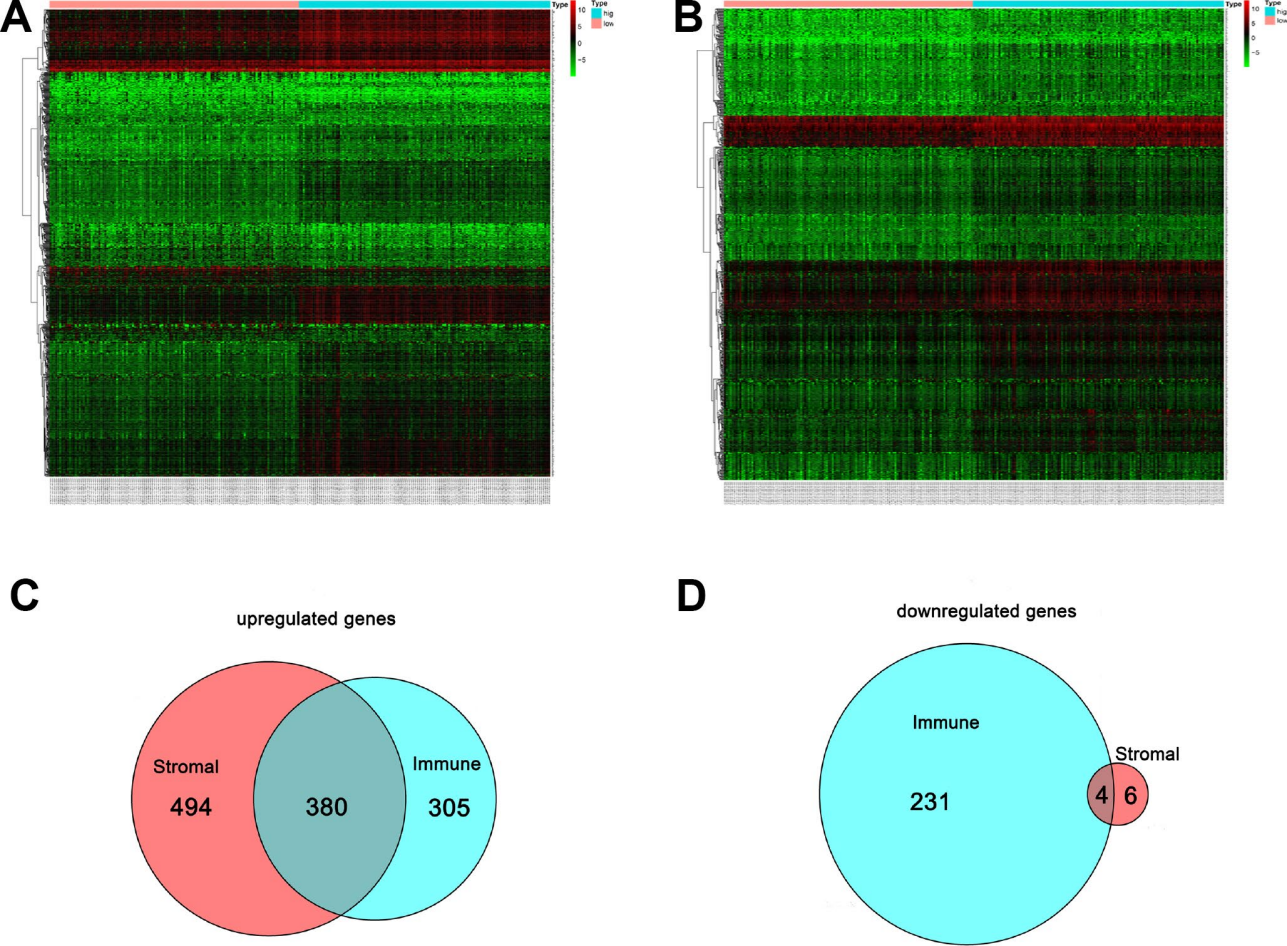
**Comparison of the gene expression profile with immune and stromal scores.** In the heat maps, genes with higher expression are shown in red, and lower expression are shown in green; genes expressed at the same level are in black. (**A**) Based on immune score comparisons, 685 genes were upregulated and 235 genes were downregulated in the high score group as compared to the low score group. (**B**) Based on stromal score comparisons, 874 genes were upregulated and 10 genes were downregulated in the high score group as compared to the low score group. (**C**) A total of 380 genes were commonly upregulated in the immune and stromal score groups. (**D**) A total of 4 genes were commonly downregulated in the immune and stromal score groups.

Figure 4A shows a heat map of 920 genes differentially expressed between patients with high or low immune scores, while Figure 4B shows a heat map for 884 genes differentially expressed between patients with high or low stromal scores. For immune scores, 685 genes were upregulated and 235 were downregulated in the high score group as compared to the low score group. For stromal scores, 874 genes were upregulated and 10 were downregulated in the high score group as compared to the low score group. Venn diagrams showed that 380 intersection genes were upregulated (Figure 4C) and 4 were downregulated (Figure 4D) in both the immune and stromal groups.

### Functional analysis of intersection genes

**Figure 5:**
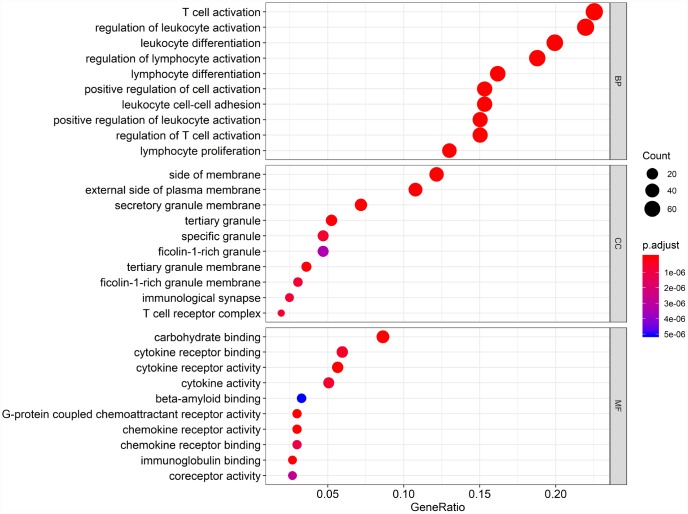
**Gene Ontology (GO) analysis of the 384 intersection genes.**

**Figure 6:**
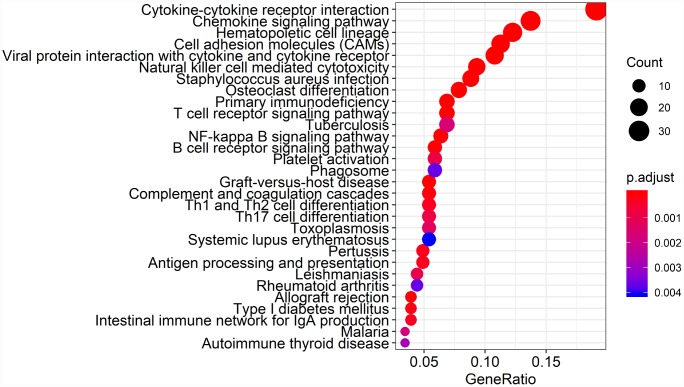
**Kyoto Encyclopedia of Genes and Genomes (KEGG) analysis of the 384 intersection genes.**

Biological enrichment analysis, including KEGG pathways and GO analyses, were performed to further evaluate the biological functions of the 384 intersection genes. These genes were mainly involved in several biological processes (BP) that are closely related to T cell activation. The cellular component (CC) process revealed that the target genes are mainly involved in the region of the cell membrane. The molecular functions (MF) process revealed that the target genes are significantly related to carbohydrate binding (Figure 5). The KEGG pathways were mainly enriched for cytokine-cytokine receptor interaction (Figure 6).

### PPI of intersection genes

To investigate hub genes among the intersection genes and develop a thorough picture of the intersection genes at the systems level, all 384 intersection genes were uploaded to the STRING database to construct a PPI network (Supplementary Figure 1). By applying the MCODE tool in Cytoscape software, the most highly connected intersection genes were identified. In total, 269 intersection genes were found to be connected with other genes. Among these, *ITGAM, PTPRC, ITGAX, TYEOBP, and C3AR1* were the top 5 with 57, 56, 45, 45, and 44 nodes, respectively. The connected nodes for each intersection gene are listed in Supplementary Table 1.

### Survival analysis of intersection genes

**Figure 7:**
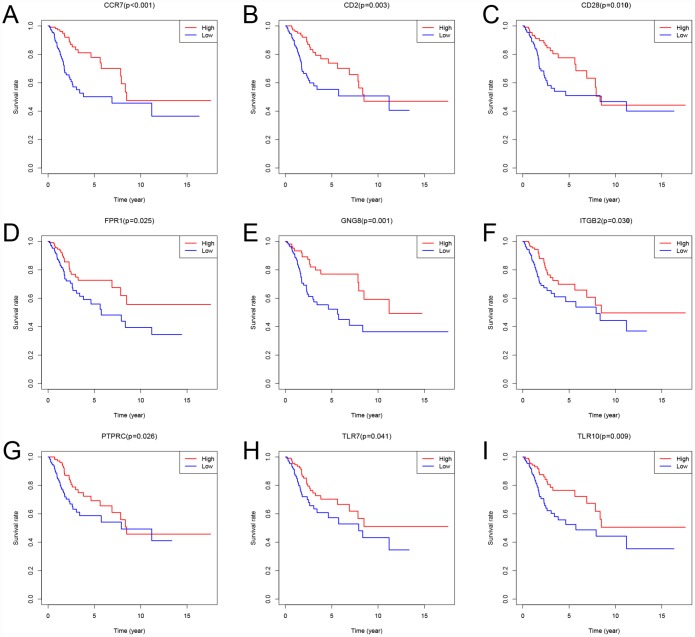
**Kaplan-Meier survival curves showing the impact of expression level of 9 random genes with overall survival.** Comparison of overall survival in the high (red line) and low (blue line) gene expression groups. P < 0.05 was used to assess differences in Log-rank test.

To assess the relationship between each intersection gene and overall survival, we constructed Kaplan-Meier survival curves using the TCGA database. Using the log-rank test, we found that among the 384 intersection genes, 149 were predictive of overall survival (P < 0.05) (Supplementary Table 2). Kaplan-Meier survival curves illustrating the effect of nine random genes on overall survival are shown in Figure 7.

## DISCUSSION

In this era of precise medicine, identification of effective biomarkers for cancer-specific prognoses is urgently needed to enhance decision making for patient management. In the present study, we used bioinformation analysis to screen for prognostic immune related genes in CSCC. The results showed that high immune scores were associated with better overall survival. Moreover, we detected 149 immune-related genes in the tumor microenvironment that may have the potential to serve as prognostic biomarkers for CSCC.

We used ESTIMATE to output immune scores, which reflected the level of immune cells infiltration into the tumor tissue [9]. We found that high immune scores were associated with better overall survival. Similarly, Heeren et al. [16] reported significantly lower CD4(+) T-cell frequencies in lymph node-positive samples than lymph node-negative ones. In addition, Punt et al. [17] reported that B cell expression of TCL1A correlated with improved survival (P = 0.007) in CSCC. These results may indicate that immune cells infiltrating tumor tissue act to inhibit cancer cells. Consistent with that idea, we observed that T4 stage and N1 stage were associated with poor overall survival in the low immune scores group but that T stage and N stage were not associated with overall survival in the high immune scores group.

Immune scores were calculated based on a comprehensive score for all genes in the tissues [9]. Although the immune score is related to prognosis, the immune-specific genes are not necessarily related to prognosis. Similarly, although the stromal score is not directly related to prognosis, this does not mean that stroma-specific genes are not associated with prognosis. We attempted to identify a group of intersection genes that were differentially expressed in both immune and stromal cells. Because the intersection genes are the most highly conserved, we suggest they are the most likely to be associated with prognosis.

Finally, 380 upregulated intersection genes and 4 downregulated intersection genes were detected between the immune and stromal score groups. These 384 intersection genes were associated with biological processes in the tumor microenvironment, including T cell activation, regulation of leukocyte activation, leukocyte differentiation, and regulation of lymphocyte activation. These processes may inhibit tumor progression and metastasis [16], which could in turn improve survival [6]. Among molecular functions, the 384 intersection genes were mainly involved in cytokine receptor binding, cytokine receptor activity, and cytokine activity. Cytokines are usually secreted in response to an activating stimulus and induce responses through binding to specific receptors on the surface of target cells [18]. It has been reported that the cytokine-cytokine receptor interaction gene set could potentially induce cancer [19]. The upregulation of genes involved in cytokine-cytokine receptor interactions were consistently detected in tumor cell lines. These genes may thus be potential biomarkers for early diagnosis. However, further investigation into the function of the 384 intersection genes is needed.

A PPI network was constructed to reveal the relationship and function of the 384 intersection genes. *ITGAM, PTPRC, ITGAX, TYEOBP,* and *C3AR1* were the top 5 genes, with 57, 56, 45, 45 and 44 nodes, respectively. Among these genes, only *PTPRC* was associated with overall survival. *PTPRC* encodes the first known and prototypical receptor-like protein tyrosine phosphatase. PTPRC enzyme functions as a central regulator of phosphotyrosine levels in hematopoietic cells by modulating the activity of Src family kinases. Its importance is highlighted by observations in both mice and humans that its absence leads to severe combined immunodeficiency, while dysregulation of its activity correlates with autoimmunity [20, 21]. It was also found that a higher percentage of tumor occupied by PTPRC+ cells was strongly associated with enhanced tumor-infiltration by Tbet+ cells and Foxp3+ cells [22]. The area occupied by preferentially type I-oriented PTPRC+ cell infiltrate was associated with longer disease-free and disease-specific survival. This suggests PTPRC is a prognostic factor for recurrence-free and disease-specific survival in CSCC. Similarly, our study revealed that high levels of *PTPRC* expression are associated with better overall survival.

In this study, *ITGAM, ITGAX, TYEOBP,* and *C3AR1* were not associated with overall survival. *ITGAM* and *ITGAX* are mainly involved in systemic lupus erythematosus [23, 24], while *TYEOBP* and *C3AR1* have been studied in the context of breast cancer [25, 26]. To the best of our knowledge, however, no association between these four genes and CSCC has been reported until now. Several genes known to be prognostic did not appear in our PPI network, which indicates that further studies are needed to identify novel prognostic genes for CSCC.

The present study identified 149 prognostic genes. Among these genes, Toll-like receptors (TLRs) are recognition receptors presented on the cell membrane and in the cytoplasm that specifically recognize pathogen-associated molecular patterns. TLRs are abundantly expressed on innate immune cells, including mast cells, dendritic cells, macrophages, endothelial cells, neutrophils, and natural killer cells [27]. Moreover, in CSCC samples, TLRs appear to regulate the local immune microenvironment [28–31]. Our study showed that high levels of TLR7 and TLR10 are associated with better overall survival. This suggests that our big data-based analysis using TCGA cohorts has prognostic value.

This study has a major limitation. The 149 prognostic genes in the tumor microenvironment were identified from TCGA database using the ESTIMATE algorithm. These genes need to be independently validated before they can be useful for evaluating the prognosis of CSCC patients. To exclude bias, we plan to assess their effectiveness in clinical experiments. We anticipate that the results of these clinical experiments will enable us to determine whether combinations of these genes are more predictive of prognosis than any of the individual genes alone.

In conclusion, this study provides a more comprehensive understanding of the tumor microenvironment as well as a list of prognostic immune-related genes in CSCC. These genes need to be investigated further to gain additional insight into the association between the tumor microenvironment and prognosis in CSCC.

## MATERIALS AND METHODS

### Data

Gene expression profiles of CSCC were downloaded from The Cancer Genome Atlas (TCGA) dataset (https://tcga-data.nci.nih.gov/tcga/). Clinical data, including age, T stage, N stage, tumor grade, and survival, were also downloaded from TCGA. Inclusion criteria were (1) pathology confirmed CSCC, (2) complete RNA expression data from the patients were available, and (3) patents were ≥ 18 years of age.

### Transcriptional expression profile

The ESTIMATE algorithm was used to calculate immune and stromal scores using the estimate package at http://r-forge.r-project.org [9]. ESTIMATE is an algorithm developed to assess the purity of cancer cells within a tumor, and the numbers of stromal and immune cells present within cancer tissues using TCGA gene expression matrix as an input. ESTIMATE outputs stromal, immune and ESTIMATE scores using the Illumina HiSeq RNA Sequencing platform from the University of North Carolina TCGA genome characterization center. Gene expression values were rank-normalized and rank-ordered. The cumulative distribution functions of the genes in the signature and the remaining genes were calculated. A statistic was calculated through integration of the difference between the cumulative distribution function, which is similar to the one used in gene set-enrichment analysis, but based on absolute expression rather than differential expression. Immune and stromal scores were obtained by applying the above method. The scores were used to reflect the level of immune cell infiltration of in tumor tissue. Similarly, stromal scores were also obtained by applying the above method and used to estimate the number of stromal cells present.

T stage, N stage, and tumor grade were analyzed and displayed according the immune, stromal and ESTIMATE score. One-way ANOVA was utilized to assess differences between groups. X-tile software was utilized to determine cut-off values for the immune score, stromal score and ESTIMATE scores. The cut-off values were then used to divide total participants to two groups [32]. The primary end point was overall survival, which was evaluated from the date of first therapy to the date of death or last follow-up.

### Differentially expressed gene analysis

Fragments Per Kilobase Million (FPKM) was used to count the reads of a fragment for paired-end RNA-seq dataset, which produced two mapped reads [33]. The first steps used to process DNA microarray were preprocessing and normalization of raw biological data, which removed bias to ensure uniformity and integrity of the data. We then performed background correction, propensity analysis, normalization and visualization output of probe data using a robust multi-array average analysis algorithm in the Limma package in R. Fold changes > 1.0 and P < 0.05 were set as the cut-offs used to screen for differentially expressed genes. Heat maps were also generated using the Limma package in R.

Intersection genes among the upregulated genes were identified as genes that are upregulated or downregulated in both high immune and stromal score groups as compared to the corresponding low score groups.

### Functional analysis

Kyoto Encyclopedia of Genes and Genomes (KEGG) analyses and Gene Ontology (GO) were used to assess the functional role of intersection genes. GO and KEGG pathway enrichment analyses were performed through the DAVID (http://david.ncifcrf.gov, Version 6.8) online analysis tool. P < 0.05 was the cut-off value.

### Protein-protein interaction (PPI) network and intersection genes

To further investigate intersection genes, a PPI network was constructed using the STRING online database (http://string-db.org) [34]. Validated interactions with a combined score > 0.7 were considered statistically significant. The network graph was visualized and analyzed using Cytoscape software (http://www.cytoscape.org/) [35]. The Cytoscape plug-in and Network Analyzer were applied to analyze the degree distribution. The functional modules of the network were detected using the Molecular Complex Detection MCODE plug-in.

### Correlation between intersection genes and overall survival

Kaplan-Meier plots were generated to illustrate the relationship between patients’ overall survival and intersection genes. Expression levels of intersection genes were identified as binary variables (high vs. low) using the median expression as the cut-off value for each intersection genes. The log-rank test was used to assess differences between survival curves.

### Statistical analysis

R software (version 3.3.3) and GraphPad Prism 5 were used construct the plots shown in the figures. Statistical analyses were conducted using R software (version 3.3.3) and SPSS 24.0 (SPSS, Inc., Chicago, IL, USA). A two-tailed P values < 0.05 were considered statistically significant.

## Supplementary Material

Supplementary Figure 1

Supplementary Table 1

Supplementary Table 2
