# Supplementary material for: Prognostic genes in the tumor microenvironment in cervical squamous cell carcinoma
Source: Aging (Albany NY). 2019 Nov 18;11(22):10154–66. doi: 10.18632/aging.102429 (PMC6914434; doi:10.18632/aging.102429)
Supplement: Supplementary Figure 1 [file aging-11-102429-s003..pdf]

SUPPLEMENTARY FIGURE

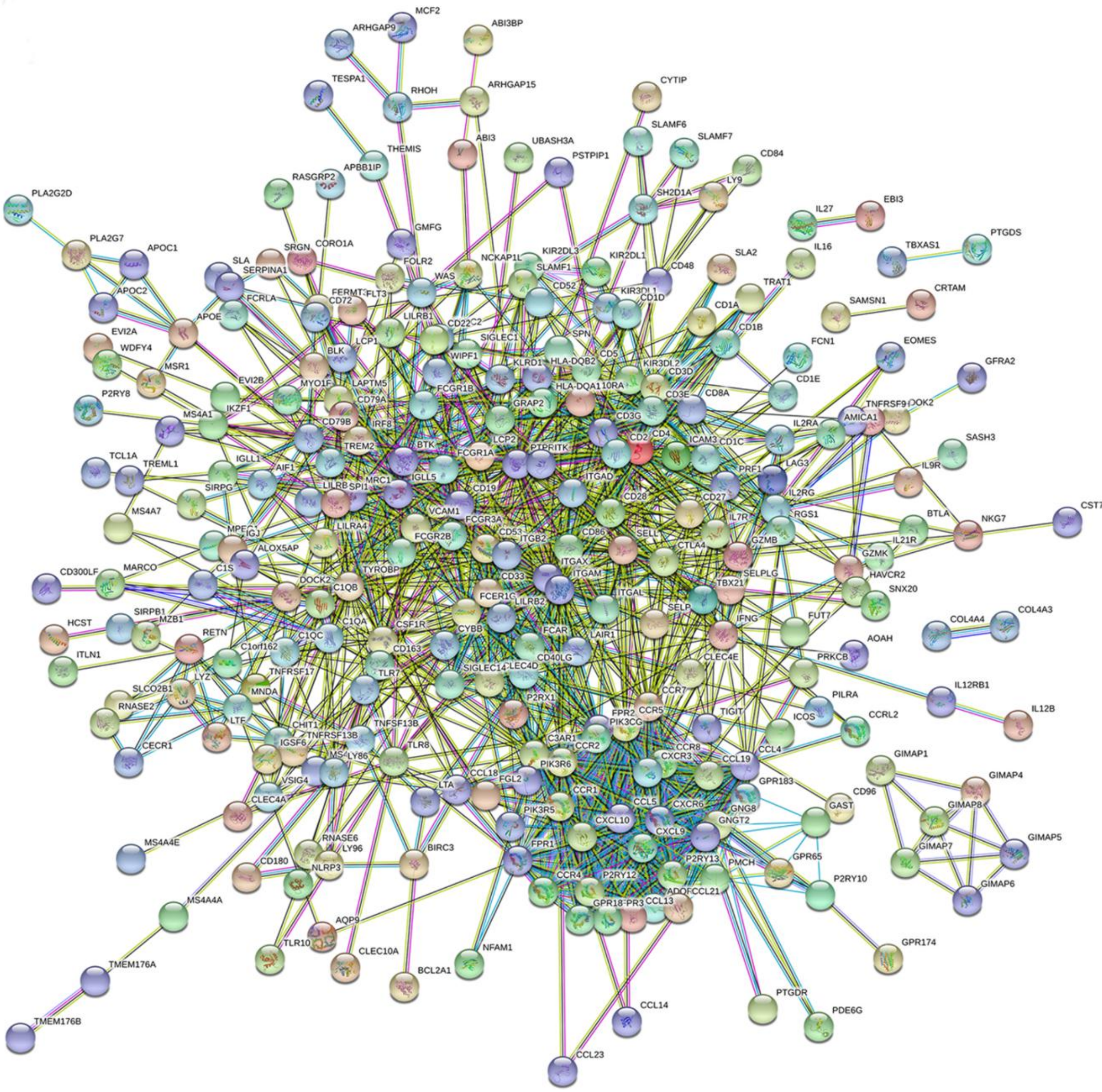

Supplementary Figure 1. Protein-protein interactions network for the 384 intersection genes. *ITGAM, PTPRC, ITGAX, TYEOBP, and C3AR1* were the top 5 genes, with 57, 56, 45, 45, and 44 nodes in the network, respectively.
