# Supplementary material for: Prognostic genes in the tumor microenvironment in cervical squamous cell carcinoma
Source: Aging (Albany NY). 2019 Nov 18;11(22):10154–66. doi: 10.18632/aging.102429 (PMC6914434; doi:10.18632/aging.102429)
Supplement: Supplementary Table 1 [file aging-11-102429-s002..docx]

| Table 1. Connected nodes of intersection genes in the protein-protein interaction network. | |
| --- | --- |
| Gene | Connected nodes |
| *ITGAM* | 57 |
| *PTPRC* | 56 |
| *ITGAX* | 45 |
| *TYROBP* | 45 |
| *C3AR1* | 44 |
| *FPR2* | 44 |
| *CCR7* | 43 |
| *CCR5* | 42 |
| *CD86* | 40 |
| *ITGB2* | 39 |
| *CD2* | 36 |
| *GNG8* | 35 |
| *GNGT2* | 35 |
| *CCR2* | 34 |
| *CXCR3* | 34 |
| *CD19* | 33 |
| *CCR1* | 32 |
| *CD28* | 31 |
| *CTLA4* | 31 |
| *FCER1G* | 31 |
| *FPR1* | 31 |
| *CCL5* | 30 |
| *CXCL10* | 30 |
| *CCL19* | 29 |
| *CCL4* | 29 |
| *PMCH* | 29 |
| *CCR8* | 28 |
| *CD3E* | 28 |
| *CSF1R* | 28 |
| *CXCL9* | 28 |
| *CXCR6* | 28 |
| *SELL* | 28 |
| *CCL21* | 27 |
| *CCR4* | 27 |
| *CD3G* | 27 |
| *GPR183* | 27 |
| *P2RY12* | 27 |
| *P2RY13* | 27 |
| *ADORA3* | 26 |
| *CCL13* | 26 |
| *FPR3* | 26 |
| *GPR18* | 26 |
| *LCP2* | 26 |
| *LILRB2* | 26 |
| *FCGR1A* | 25 |
| *CD3D* | 24 |
| *CD5* | 24 |
| *CD53* | 24 |
| *CD33* | 23 |
| *CD4* | 23 |
| *GZMB* | 22 |
| *IFNG* | 22 |
| *IGLL5* | 21 |
| *ITGAL* | 21 |
| *C1QB* | 20 |
| *CD40LG* | 20 |
| *SPI1* | 20 |
| *BTK* | 19 |
| *GRAP2* | 19 |
| *PRF1* | 19 |
| *VCAM1* | 19 |
| *C1QA* | 18 |
| *CD163* | 18 |
| *CD8A* | 18 |
| *CYBB* | 18 |
| *IL7R* | 18 |
| *TBX21* | 18 |
| *CD27* | 17 |
| *FCGR3A* | 17 |
| *CD79A* | 16 |
| *FCAR* | 16 |
| *FCGR2B* | 16 |
| *LAIR1* | 16 |
| *TLR8* | 16 |
| *CLEC4D* | 15 |
| *ITK* | 15 |
| *CD79B* | 14 |
| *IL2RG* | 14 |
| *LAPTM5* | 14 |
| *TREM2* | 14 |
| *CCL18* | 13 |
| *HLA-DQA1* | 13 |
| *HLA-DQB2* | 13 |
| *KLRD1* | 13 |
| *MRC1* | 13 |
| *MS4A6A* | 13 |
| *P2RX1* | 13 |
| *SIGLEC14* | 13 |
| *TLR7* | 13 |
| *LY86* | 12 |
| *WAS* | 12 |
| *C1QC* | 11 |
| *CD1C* | 11 |
| *SPN* | 11 |
| *IL2RA* | 10 |
| *IRF8* | 10 |
| *KLRC2* | 10 |
| *BLK* | 9 |
| *CD1D* | 9 |
| *CD22* | 9 |
| *CD48* | 9 |
| *DOCK2* | 9 |
| *IKZF1* | 9 |
| *LAG3* | 9 |
| *MNDA* | 9 |
| *RETN* | 9 |
| *CD1B* | 8 |
| *CD52* | 8 |
| *ITGAD* | 8 |
| *NCKAP1L* | 8 |
| *APOE* | 7 |
| *CD1A* | 7 |
| *FCGR1B* | 7 |
| *FERMT3* | 7 |
| *LCP1* | 7 |
| *LYZ* | 7 |
| *P2RY10* | 7 |
| *PIK3CG* | 7 |
| *SH2D1A* | 7 |
| *VSIG4* | 7 |
| *AIF1* | 6 |
| *FLT3* | 6 |
| *GAST* | 6 |
| *GPR65* | 6 |
| *GZMH* | 6 |
| *GZMK* | 6 |
| *HAVCR2* | 6 |
| *IGJ* | 6 |
| *IGSF6* | 6 |
| *KIR2DL1* | 6 |
| *KIR2DL3* | 6 |
| *LY96* | 6 |
| *PIK3R6* | 6 |
| *RGS1* | 6 |
| *SELP* | 6 |
| *SELPLG* | 6 |
| *SLAMF1* | 6 |
| *TIGIT* | 6 |
| *TNFRSF9* | 6 |
| *TNFSF13B* | 6 |
| *BIRC3* | 5 |
| *CD1E* | 5 |
| *CD72* | 5 |
| *CHIT1* | 5 |
| *GIMAP4* | 5 |
| *GIMAP7* | 5 |
| *GIMAP8* | 5 |
| *IL10RA* | 5 |
| *KIR3DL1* | 5 |
| *KIR3DL2* | 5 |
| *LILRB1* | 5 |
| *MPEG1* | 5 |
| *NKG7* | 5 |
| *PIK3R5* | 5 |
| *SIGLEC1* | 5 |
| *TRAT1* | 5 |
| *ALOX5AP* | 4 |
| *C1orf162* | 4 |
| *C1S* | 4 |
| *CECR1* | 4 |
| *EOMES* | 4 |
| *EVI2B* | 4 |
| *FCRLA* | 4 |
| *FGL2* | 4 |
| *GIMAP5* | 4 |
| *GIMAP6* | 4 |
| *IGLL1* | 4 |
| *LTA* | 4 |
| *LTF* | 4 |
| *MS4A1* | 4 |
| *MYO1F* | 4 |
| *PLA2G7* | 4 |
| *PRKCB* | 4 |
| *RHOH* | 4 |
| *RNASE2* | 4 |
| *SLA2* | 4 |
| *TNFRSF17* | 4 |
| *WIPF1* | 4 |
| *APOC1* | 3 |
| *APOC2* | 3 |
| *BTLA* | 3 |
| *CCRL2* | 3 |
| *CLEC4A* | 3 |
| *CORO1A* | 3 |
| *DOK2* | 3 |
| *FOLR2* | 3 |
| *FUT7* | 3 |
| *GIMAP1* | 3 |
| *ICAM3* | 3 |
| *LILRA4* | 3 |
| *LILRB4* | 3 |
| *LY9* | 3 |
| *MARCO* | 3 |
| *MS4A7* | 3 |
| *NLRP3* | 3 |
| *SERPINA1* | 3 |
| *SRGN* | 3 |
| *TNFRSF13B* | 3 |
| *ABI3* | 2 |
| *AQP9* | 2 |
| *ARHGAP15* | 2 |
| *CCL14* | 2 |
| *CCL23* | 2 |
| *CD180* | 2 |
| *CD84* | 2 |
| *GMFG* | 2 |
| *ICOS* | 2 |
| *IL12RB1* | 2 |
| *IL21R* | 2 |
| *MS4A4A* | 2 |
| *MSR1* | 2 |
| *MZB1* | 2 |
| *NFAM1* | 2 |
| *PDE6G* | 2 |
| *PSTPIP1* | 2 |
| *PTGDR* | 2 |
| *RNASE6* | 2 |
| *SIRPG* | 2 |
| *SLA* | 2 |
| *SLAMF6* | 2 |
| *SLAMF7* | 2 |
| *SLCO2B1* | 2 |
| *THEMIS* | 2 |
| *TLR10* | 2 |
| *TMEM176A* | 2 |
| *TREML1* | 2 |
| *ABI3BP* | 1 |
| *AMICA1* | 1 |
| *AOAH* | 1 |
| *APBB1IP* | 1 |
| *ARHGAP9* | 1 |
| *BCL2A1* | 1 |
| *CD300LF* | 1 |
| *CD96* | 1 |
| *CLEC10A* | 1 |
| *CLEC4E* | 1 |
| *COL4A3* | 1 |
| *COL4A4* | 1 |
| *CRTAM* | 1 |
| *CST7* | 1 |
| *CYTIP* | 1 |
| *EBI3* | 1 |
| *EVI2A* | 1 |
| *FCN1* | 1 |
| *GFRA2* | 1 |
| *GPR174* | 1 |
| *HCST* | 1 |
| *IL12B* | 1 |
| *IL16* | 1 |
| *IL27* | 1 |
| *IL9R* | 1 |
| *ITLN1* | 1 |
| *MCF2* | 1 |
| *MS4A4E* | 1 |
| *P2RY8* | 1 |
| *PILRA* | 1 |
| *PLA2G2D* | 1 |
| *PTGDS* | 1 |
| *RASGRP2* | 1 |
| *SAMSN1* | 1 |
| *SASH3* | 1 |
| *SIRPB1* | 1 |
| *SNX20* | 1 |
| *TBXAS1* | 1 |
| *TCL1A* | 1 |
| *TESPA1* | 1 |
| *TMEM176B* | 1 |
| *UBASH3A* | 1 |
| *WDFY4* | 1 |
