# Supplementary material for: Prognostic genes in the tumor microenvironment in cervical squamous cell carcinoma
Source: Aging (Albany NY). 2019 Nov 18;11(22):10154–66. doi: 10.18632/aging.102429 (PMC6914434; doi:10.18632/aging.102429)
Supplement: Supplementary Table 2 [file aging-11-102429-s001..docx]

| Table 2. Relationships between intersection genes and overall survival of cervical squamous cell carcinoma. | |
| --- | --- |
| Gene | P for overall survival |
| *ABCB1* | 0.023 |
| *AC136428.1* | 0.021 |
| *AOAH* | 0.033 |
| *ARHGAP9* | 0.040 |
| *ARHGAP15* | 0.030 |
| *BTK* | 0.046 |
| *BTLA* | 0.015 |
| *CASQ1* | 0.022 |
| *CCL5* | 0.025 |
| *CCL19* | 0.017 |
| *CCR2* | 0.009 |
| *CCR4* | 0.005 |
| *CCR7* | <0.001 |
| *CD1A* | 0.007 |
| *CD1B* | 0.012 |
| *CD1C* | 0.003 |
| *CD1E* | <0.001 |
| *CD2* | 0.003 |
| *CD3D* | 0.009 |
| *CD3E* | 0.020 |
| *CD3G* | 0.014 |
| *CD5* | <0.001 |
| *CD8A* | 0.013 |
| *CD19* | 0.038 |
| *CD27* | <0.001 |
| *CD28* | 0.010 |
| *CD33* | 0.047 |
| *CD37* | 0.034 |
| *CD40LG* | 0.033 |
| *CD48* | 0.013 |
| *CD72* | 0.044 |
| *CD79A* | 0.005 |
| *CD79B* | 0.029 |
| *CD300LF* | 0.010 |
| *CELF2* | 0.049 |
| *CERKL* | 0.049 |
| *CHIT1* | 0.021 |
| *CLEC4E* | 0.034 |
| *CRTAM* | 0.050 |
| *CST7* | 0.001 |
| *CTLA4* | 0.011 |
| *CXCL9* | 0.027 |
| *CXCR3* | 0.031 |
| *CXCR6* | 0.005 |
| *CXorf65* | 0.003 |
| *CYTIP* | 0.008 |
| *DOCK2* | 0.022 |
| *FCRL3* | 0.008 |
| *FCRL5* | 0.027 |
| *FERMT3* | 0.024 |
| *FGL2* | 0.037 |
| *FPR1* | 0.025 |
| *FUT7* | 0.003 |
| *GNG8* | 0.001 |
| *GPR171* | 0.020 |
| *GRAP2* | 0.008 |
| *GZMH* | 0.014 |
| *GZMM* | 0.043 |
| *HAVCR2* | 0.022 |
| *HK3* | 0.018 |
| *HLA.DQA1* | 0.043 |
| *ICOS* | 0.045 |
| *IGLL5* | 0.047 |
| *IGSF6* | 0.010 |
| *IKZF1* | 0.031 |
| *IL10RA* | 0.009 |
| *IL12B* | 0.005 |
| *IL12RB1* | 0.011 |
| *IL16* | 0.009 |
| *IL21R* | 0.018 |
| *ITGAD* | 0.041 |
| *ITGAL* | 0.018 |
| *ITGB2* | 0.030 |
| *ITK* | 0.003 |
| *ITM2A* | 0.048 |
| *JAKMIP1* | 0.036 |
| *JAML* | 0.029 |
| *JCHAIN* | 0.004 |
| *KCNA3* | 0.004 |
| *KLHL6* | 0.012 |
| *KLRC2* | 0.022 |
| *LAX1* | 0.020 |
| *LCP2* | 0.039 |
| *LILRA4* | <0.001 |
| *LILRB1* | 0.028 |
| *LILRB4* | 0.008 |
| *LTA* | 0.046 |
| *LY9* | <0.001 |
| *MPEG1* | 0.028 |
| *MYO1F* | 0.009 |
| *NFAM1* | 0.017 |
| *P2RX1* | 0.004 |
| *P2RY8* | 0.002 |
| *P2RY10* | 0.021 |
| *P2RY13* | 0.007 |
| *PEAK3* | 0.029 |
| *PIK3R5* | 0.022 |
| *PILRA* | 0.036 |
| *PLA2G2D* | 0.002 |
| *PLA2G7* | 0.004 |
| *PLD4* | 0.008 |
| *PRAM1* | 0.010 |
| *PRKCB* | 0.033 |
| *PSTPIP1* | 0.026 |
| *PTGDS* | 0.042 |
| *PTPRC* | 0.026 |
| *PYHIN1* | 0.013 |
| *RAB33A* | 0.015 |
| *RASAL3* | 0.043 |
| *RASGRP2* | 0.013 |
| *RCSD1* | 0.026 |
| *RETN* | 0.047 |
| *RHOH* | 0.013 |
| *RIPOR2* | 0.010 |
| *SASH3* | 0.041 |
| *SCIMP* | 0.016 |
| *SCML4* | 0.002 |
| *SCUBE1* | 0.011 |
| *SELL* | <0.001 |
| *SELPLG* | 0.007 |
| *SH2D1A* | 0.002 |
| *SIGLEC1* | 0.033 |
| *SIRPG* | 0.010 |
| *SIT1* | 0.004 |
| *SLA* | 0.012 |
| *SLAMF1* | <0.001 |
| *SLAMF6* | 0.001 |
| *SLAMF7* | 0.039 |
| *SNX20* | 0.009 |
| *SPIB* | <0.001 |
| *SPOCK2* | 0.029 |
| *TBC1D10C* | 0.018 |
| *TESPA1* | 0.025 |
| *THEMIS* | 0.016 |
| *TIFAB* | 0.021 |
| *TIGIT* | 0.007 |
| *TIMD4* | 0.038 |
| *TLR7* | 0.041 |
| *TLR10* | 0.009 |
| *TMIGD3* | 0.016 |
| *TNFAIP8LA* | 0.038 |
| *TRAT1* | 0.020 |
| *TREM2* | 0.038 |
| *TREML1* | 0.019 |
| *TRPV2* | 0.037 |
| *UBASH3A* | 0.003 |
| *WAS* | 0.018 |
| *WDFY4* | 0.023 |
| *ZNF831* | <0.001 |
